# Exploratory application of a cannulation model in recently weaned pigs to monitor longitudinal changes in the enteric microbiome across varied porcine reproductive and respiratory syndrome virus (PRRSV) infection statuses

**DOI:** 10.3389/fvets.2024.1422012

**Published:** 2024-07-19

**Authors:** Tanja Opriessnig, Patrick Halbur, Jenna Bayne, Gaurav Rawal, Hao Tong, Kathy Mou, Ganwu Li, Danyang Zhang, Jianqiang Zhang, Adrian Muwonge

**Affiliations:** ^1^Department of Vaccines and Diagnostics, Moredun Research Institute, Penicuik, United Kingdom; ^2^Department of Veterinary Diagnostic and Production Animal Medicine, College of Veterinary Medicine, Iowa State University, Ames, IA, United States; ^3^Department of Clinical Sciences, College of Veterinary Medicine, Auburn University, Auburn, AL, United States; ^4^The Digital One Health Laboratory, Roslin Institute and The Royal (Dick) School of Veterinary Studies, University of Edinburgh, Midlothian, United Kingdom

**Keywords:** pigs, porcine reproductive and respiratory syndrome virus, antibiotics, probiotics, vaccination, ileum microbiome

## Abstract

**Introduction:**

The enteric microbiome and its possible modulation to improve feed conversion or vaccine efficacy is gaining more attention in pigs. Weaning pigs from their dam, along with many routine procedures, is stressful. A better understanding of the impact of this process on the microbiome may be important for improving pig production. The objective of this study was to develop a weaner pig cannulation model, thus allowing ileum content collection from the same pig over time for 16S rRNA sequencing under different porcine reproductive and respiratory syndrome virus (PRRSV) infection statuses.

**Methods:**

A total of 15 3-week-old pigs underwent abdominal surgery and were fitted with an ileum cannula, with ileum contents collected over time. In this pilot study, treatment groups included a NEG-CONTROL group (no vaccination, no PRRSV challenge), a POS-CONTROL group (no vaccination, challenged with PRRSV), a VAC-PRRSV group (vaccinated, challenged with PRRSV), a VAC-PRO-PRRSV group (vaccinated, supplemented with a probiotic, challenged with PRRSV), and a VAC-ANTI-PRRSV group (vaccinated, administered an antibiotic, challenged with PRRSV). We assessed the microbiome over time and measured anti-PRRSV serum antibodies, PRRSV load in serum and nasal samples, and the severity of lung lesions.

**Results:**

Vaccination was protective against PRRSV challenge, irrespective of other treatments. All vaccinated pigs mounted an immune response to PRRSV within 1 week after vaccination. A discernible impact of treatment on the diversity, structure, and taxonomic abundance of the enteric microbiome among the groups was not observed. Instead, significant influences on the ileum microbiome were observed in relation to time and treatment.

**Discussion:**

The cannulation model described in this pilot study has the potential to be useful in studying the impact of weaning, vaccination, disease challenge, and antimicrobial administration on the enteric microbiome and its impact on pig health and production. Remarkably, despite the cannulation procedures, all vaccinated pigs exhibited robust immune responses and remained protected against PRRSV challenge, as evidenced by the development of anti-PRRSV serum antibodies and viral shedding data.

## 1 Introduction

In recent years, substantial advances in understanding the gut microbiome have been facilitated by highly efficient sequencing tools (1–3). The link between the gut microbiome and health or disease is evident (4, 5). Often, parenterally administered attenuated porcine reproductive and respiratory syndrome virus (PRRSV) vaccines have less than desired efficacy under field conditions. Research in human vaccinology indicated that probiotic bacteria modulate both innate and adaptive immunity in the host (6, 7). Gut microbes have been suggested to support immune responses against viral infections by facilitating the processing and secretion of proinflammatory cytokines. In humans, probiotics are believed to have a potential influence on the response to influenza vaccination, leading to recommendations for dietary changes before the scheduled vaccinations (8, 9). Commonly, in pig production, pigs are weaned from their dam at 3–4 weeks of age and co-mingled with other litters and administered vaccines (10).

The potential benefits of gut bacteria may also be affected by the prophylactic administration of antimicrobials to pigs at the time of weaning. In addition, weaning is known to induce “dysbiosis” of the gut microbiota (11).

Currently, microbiome studies in pigs are often limited to the analysis of rectal swabs from pigs in the field with unknown disease or immune status. Previously, we studied the microbiome of pigs experimentally infected with *Lawsonia intracellularis* and treated with different types of probiotics (12). For the 16S rRNA sequencing, we used ileum samples, which required us to euthanize the pigs. The obtained results indicated significant differences in microbiome diversity across different treatment groups (12). However, the terminal study offered only a single time point glance at possible differences among treatment groups, which was associated with clinical differences among treatment groups. Identifying a way to investigate the enteric microbiome in pigs over time to assess the impact of vaccination or other treatments would be valuable.

The objectives of this pilot study were to develop a model (1) to investigate the effect of the administration of probiotics at the time of parenteral PRRSV vaccination on PRRSV vaccine efficacy (viremia, antibody response, clinical outcomes) and (2) to investigate the gut microbiome in these pigs over time using a cannulation approach followed by characterization of the bacterial population using 16s rRNA sequencing.

## 2 Materials and methods

### 2.1 Animal approval

Institutional Review Board Statement: The study was conducted according to the guidelines of the Declaration of Helsinki and approved by the Iowa State University Institutional Animal Care and Use Committee (Approval number IACUC-21-031; Date of approval: 05-April-2021) and by the Iowa State University IBC Committee (Approval number IBC 21-019; Date of approval: 6-April-2021). Environmental enrichment was provided, and independent veterinarians, not part of the research team, assessed the pigs and made decisions on welfare and euthanasia.

### 2.2 Pigs and housing

At 3 weeks of age, 15 conventional pigs were purchased from a specific pathogen-free herd, free of PRRSV, influenza A virus, and *Mycoplasma hyopneumoniae* based on monthly testing using serology and pooled PCR tests. The pigs were housed in the Livestock Infectious Disease Isolation Facility (LIDIF) at Iowa State University. Initially, all pigs were kept in one large room with five pens. The pens were placed directly on a concrete floor, with each pen enclosed by galvanized steel gates (~2 × 3 m). Each pen had a nipple drinker and a self-feeder. The pigs were offered an age-appropriate pelleted diet free of Antibiotics (Heartland Co-Op, Prairie City, IA, USA). Shortly before the pigs were vaccinated with a commercially modified live PRRSV vaccine strain, the two non-vaccinated groups (NEG-CONTROL and POS-CONTROL pigs) were moved to another room that contained two pens as described above. Before being challenged with the PRRSV strain, the POS-CONTROL pigs were moved to a separate room.

### 2.3 Experimental design

Upon arrival at the research facility, the pigs were randomly allocated to five different treatment groups (Table 1), including a NEG-CONTROL group (no vaccination, no PRRSV challenge), a POS-CONTROL group (no vaccination, challenged with PRRSV), a VAC-PRRSV group (vaccinated and challenged with PRRSV), a VAC-PRO-PRRSV group (vaccinated, supplemented with an oral probiotic every day from 19 days before vaccination until study termination, and challenged with PRRSV), and a VAC-ANTI-PRRSV group (vaccinated, administered a systemic antibiotic 3 days before vaccination, and challenged with PRRSV).

**Table 1:** Experimental design.

**Group**	**Pig #**	**Treatment**	**Vaccination**	**Challenge**
NEG-CONTROL	3	-	-	-
POS-CONTROL	3	-	-	PRRSV
VAC-ANTI-PRRSV	3	Antibiotic	Yes	PRRSV
VAC-PRO-PRRSV	3	Probiotic	Yes	PRRSV
VAC-PRRSV	3	-	Yes	PRRSV

**Figure 1:**
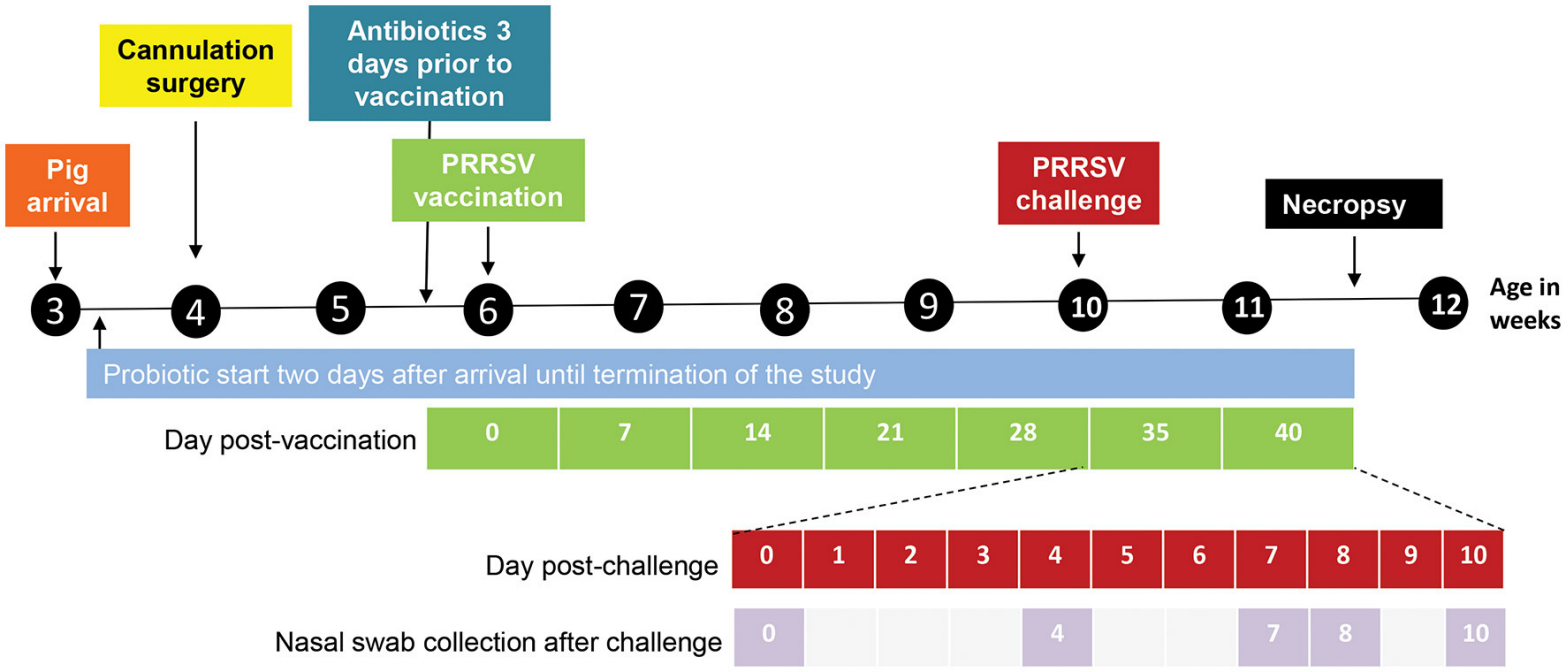
Design and outline of the different experimental steps from pig arrival until necropsy. The days post-vaccination are shown in green. The days post challenge (shown in red) and the nasal swab collection points (shown in lilac) are outlined below the timeline in an expanded view.

After an acclimation period, all pigs underwent surgery at 4 weeks of age (Figure 1) to place a stainless steel cannula into the terminal ileum, with a port on the outside of the abdominal wall to access the ileum contents. At 6 weeks of age, pigs in the VAC-PRRSV, VAC-ANTI-PRRSV, and VAC-PRO-PRRSV groups were vaccinated against PRRSV using a parenteral commercial modified live virus vaccine (Ingelvac^®^ PRRS MLV, Boehringer Ingelheim, St. Joseph MO, USA). At 10 weeks of age, with the exception of pigs in the NEG-CONTROL group, pigs in all other groups were challenged with a wild-type PRRSV strain that was administered intranasally. All pigs were euthanized and necropsied 10 days later (Figure 1).

### 2.4 Study treatments

#### 2.4.1 Probiotic treatment

Each pig in the VAC-PRO-PRRSV group received probiotics (1 g/pig/day) orally, starting 2 days after arrival (Supplementary Figure S1). The probiotic contained 25% *Bacillus subtilis*, 25% *Bacillus amyloliquefaciens*, and 50% *Enterococcus faecium* (CH3, batch 21CC0601, lot 707756, Chr. Hansen, A/S, Hørsholm, Denmark). To be consistent with the feeding routine, the pigs were fed every day between 12:00 and 15:00, except for the day of surgery when they were fed after recovery. Initially, the probiotic was mixed with Pedialyte (Abbott, Abbott Park, IL, USA) via oral lavage. In brief, each pig was picked up and placed in an upright sitting position, with extra care handling them post-operatively, and fed the solution. The probiotic carrier used was CaCO_3_, which will not dissolve even if shaken and typically remains at the bottom of the liquid. This is not considered a problem as the spores are in the liquid phase. Pigs tolerated feeding in this position moderately well with minimal waste. However, catching the pigs for this procedure became stressful as they got older. From 3 days prior to vaccination onwards, the pigs were given cereal (Captain Crunch^®^) mixed with the probiotic and the Pedialyte, which was then placed on top of their normal feed. This method was less stressful on the pigs and led to less waste, as the pigs would eat the entirety of the cereal, and any waste liquid would be consumed via their regular feed. All other groups of pigs were also given the cereal/Pedialyte mixture without probiotics.

#### 2.4.2 Antimicrobial treatment

Each VAC-ANTI-PRRSV pig received Excede^®^ for swine (Zoetis, expiration date: 12-2021; Lot ID: 408011) intramuscularly once at 3 days before vaccination according to the manufacturer's instructions (a single dose in the neck at a dosage of 2.27 mg ceftiofur equivalents/lb body weight). This ready-to-use formulation contains the crystalline-free acid of ceftiofur, which is a broad-spectrum cephalosporin antibiotic that is active against gram-positive and gram-negative bacteria including ß-lactamase-producing strains, and is effective for 7 days.

#### 2.4.3 Vaccination

When the pigs were 6 weeks old, groups VAC-PRRSV, VAC-PRO-PRRSV, and VAC-ANTI-PRRSV were vaccinated with a commercial PRRSV vaccine (Ingelvac PRRS^®^ MLV, Boehringer Ingelheim, serial number: 2451391A, expiration date: 17-Aug-2022) according to the manufacturer's instructions. In brief, the vaccine was reconstituted immediately before the planned vaccination, and each pig received 2 ml of the vaccine via intramuscular injection into the neck area using a hypodermic needle (23 gauge × 1/3 in.).

### 2.5 Clinical monitoring

Pigs were weighed on arrival at 3 weeks of age, at PRRSV vaccination at 6 weeks of age, and at necropsy at 12 weeks of age. Average daily weight gain (ADG) was calculated. After surgery, all pigs were monitored for signs of clinical disease daily. Specifically, pigs were observed for the following: fecal consistency (0 = solid; 1 = semisolid; 2 = pasty; 3 = unformed; and 4 = profuse liquid) (13); respiratory score (0 = normal; 1 = mild dyspnea and/or tachypnea when stressed; 2 = mild dyspnea and/or tachypnea when at rest; 3 = moderate dyspnea and/or tachypnea when stressed; 4 = moderate dyspnea and/or tachypnea at rest; 5 = severe dyspnea and/or tachypnea when stressed; and 6 = severe dyspnea and/or tachypnea when at rest) (14); behavior (0 = normal; 1 = depressed or listless but still standing; 3 = depressed and recumbent); and body condition score (0 = normal; 1 = mild-to-moderate gaunt; 3 = severely gaunt). In addition, rectal temperatures were recorded on pigs if there were concerns from animal caretakers or staff. Surgical wound healing was checked daily, monitoring for heat, swelling, pain on palpation, discharge, or dehiscence as well as position and patency of cannula. Defecation, abdominal distention, vomiting, diarrhea, or other signs of abdominal distress were monitored and recorded. Cannulas initially were opened daily to confirm that digesta was flowing correctly, cannulas and wounds were periodically cleaned as needed, and wounds were treated with 1% silver sulfadiazine (SSD, Flammazine) cream (Dr. Reddy's Laboratories, LA, USA) to support healing.

### 2.6 Cannulation surgery of the pigs

In previous publications, duodenal cannulation surgery has been described in detail (15–17). We obtained T-shaped cannulas (Supplementary Figure S2) from a supplier (Kremer Precision, LLC, Phoenix, AZ, USA). Specifically, the cannula barrel length was 4 cm with the distal 2.5 cm threaded on the outside. The cannula barrel's inner diameter was 1.3 cm. Details of the surgical procedure are provided in Supplementary Information S3.

### 2.7 Surgery practice on a dead pig

**Figure 2:**
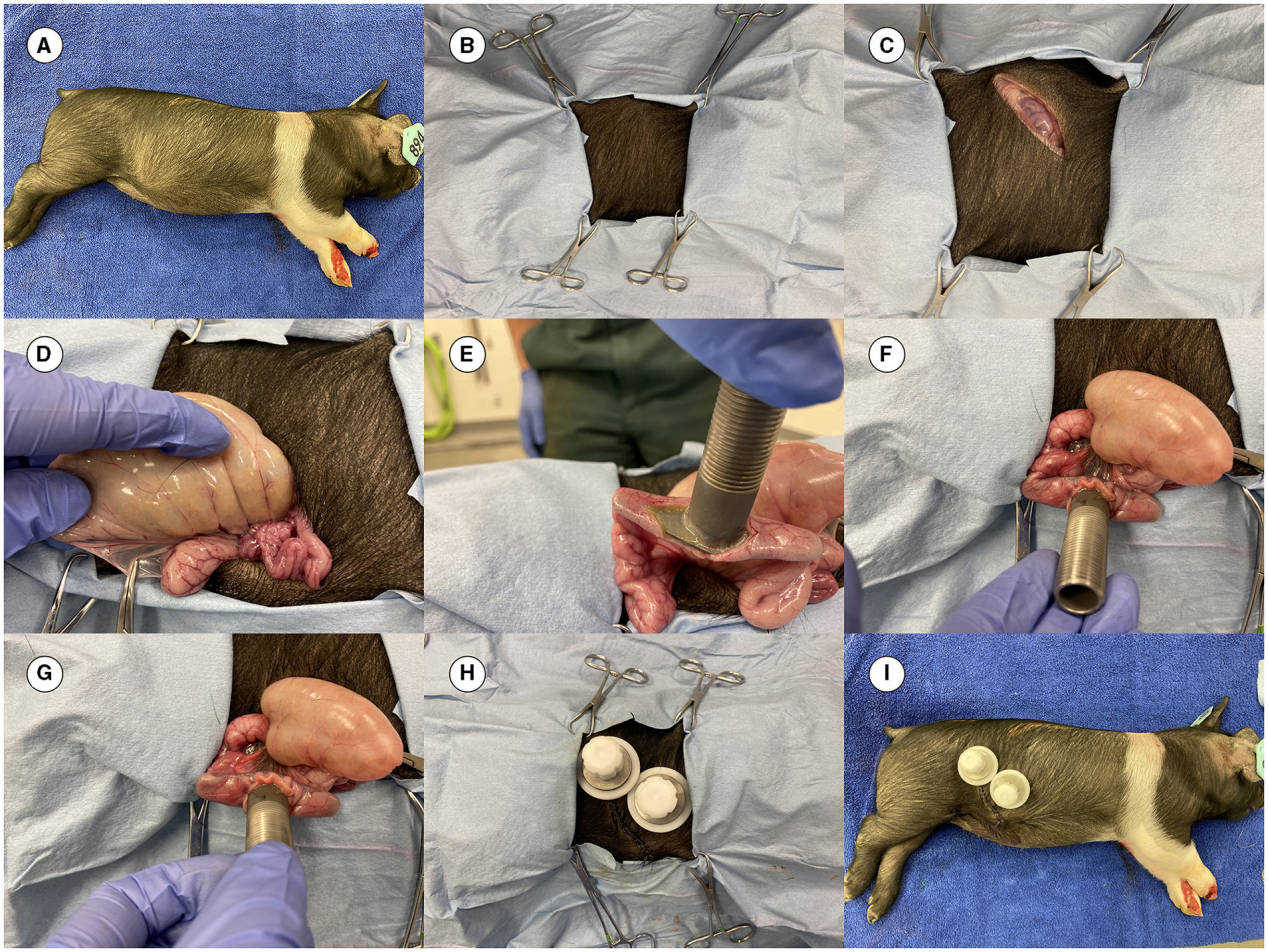
Images from the surgery. **(A)** Dead 4-week-old pig prepared for practicing the cannulation surgery. **(B)** Pig covered with surgical drapes having an opening for the incision. **(C)** Initial incision completed. **(D)** Ileum out of the abdominal cavity to locate the site for placing the cannula. **(E)** Cannula inserted before placement of the purse string suture. **(F)** Canula at the site of surgery. **(G)** Cannula is fixed in the ileum. **(H)** Final view of the placed cannula (both an ileal and a cecal cannula were inserted in the pig in this practice session). **(I)** The final complete look of the cannulas.

Before live pig cannulation, the surgical team practiced the procedure on a dead pig to comply with the Replacement, Reduction, and Refinement (3R) principle and to avoid unnecessary suffering of pigs. Once the surgical team (under the leadership of JB, an experienced surgeon) was satisfied with the surgical procedure, they proceeded to perform surgery on live pigs. More details on the surgery methods have been previously published (15, 16, 18, 19). Images from the cannulation surgery in this study are shown in Figure 2.

### 2.8 Transport to the surgery facility and pre-operative procedures

On the day of surgery, the pigs were allowed to fast overnight but were provided water. The surgeries were conducted in the surgical suite of the Food Animal and Camelid Hospital of the Iowa State University College of Veterinary Medicine. The pigs were transported in dog carriers in an enclosed van from the LIDIF to the surgery suite with a travel time of ~5 min. Upon arrival, the pigs were placed under general anesthesia and positioned in left lateral recumbency. Preoperative medications included Naxcel^®^ (ceftiofur sodium, Zoetis, Kalamazoo, MI, USA), 2.2 mg/kg, IM) and Banamine^®^-S (flunixin meglumine, Merck Animal Health, Madison, NJ, USA, 2.2 mg/kg, IM). The right flank, from the level of the stifle to the last intercostal space cranially and from the ventral to the transverse processes dorsally, was clipped and aseptically prepared with chlorhexidine and 70% isopropyl alcohol. The surgical site was draped in a routine aseptic manner. The surgeon surgically scrubbed their hands and arms using 4% chlorhexidine scrub, followed by Avagard™ (Chlorhexidine Gluconate 1% Solution and Ethyl Alcohol 61% w/w (3M Health Care, St. Paul, MN, USA), and then were aseptically gowned. Between surgeries, following the doffing of the surgical gown and gloves, Avagard™ alone was utilized before donning a new sterile surgical gown and gloves. New sterile packs and surgical instruments were utilized for each pig.

### 2.9 Challenge

For the PRRSV challenge, PRRSV strain VR2332 (NCBI: txid300559) was propagated on MARC-145 cells for three passages to a titer of 10^5.25^ 50% tissue culture infectious dose (TCID50) per ml. Each pig was infected using 5 ml of the PRRSV stock administered intranasally by slowly dripping 2.5 ml of the virus stock into each nostril. The PRRSV challenge was done 4 weeks after vaccination.

### 2.10 Sample collection

Blood samples were collected every week in vacutainer tubes before the challenge and at 3 and 6 days after the challenge (dpc), spun down, and serum was aliquoted into 2-ml tubes. In addition, nasal swabs were collected. In brief, sterile polyester-tipped swabs (Puritan^®^, Catalog No. 10805-165, Puritan Medical Products Co., Guilford, ME, USA) were inserted into each nostril, rotated 3–4 times, and placed in a 5-ml falcon tube containing 1 ml saline. The serum and the nasal swabs were stored at −70°C for further testing. Ileum content was collected once a week by restraining the pigs, unscrewing the cannula cap, and allowing the intestinal content to flow out of the cannula briefly before collecting fresh contents in a 50 ml tube, which was later allocated to small snap-top tubes and frozen at −70°C.

### 2.11 Serum and nasal swab analysis

Serum samples were tested by a commercial indirect PRRSV enzyme-linked immunosorbent assay (IDEXX PRRS X3 Ab Test; IDEXX Inc). A sample was considered positive when the sample-to-positive (S/P) value was equal to or >0.4. Serum (PRRSV viremia) and nasal swabs (PRRSV shedding) were tested using a commercial real-time PCR for the presence and quantity of PRRSV RNA. Nucleic acids were extracted from serum samples and nasal swabs using the MagMAX™ Pathogen RNA/DNA kit (Thermo Fisher Scientific) and a Kingfisher Flex instrument (Thermo Fisher Scientific) following the manufacturer's instructions. For each sample, 100 μl of the nucleic acids were eluted into 90 μl of elution buffer as described (20). A quantitative reverse transcription (RT) PCR was performed using the Commercial PRRSV screening RT-PCR, VetMAX™ PRRSV NA&EU Reagent (Thermo Fisher Scientific). A total of 8 μl nucleic acid extract was included in the final 20 μl PCR reaction. Amplification reactions were performed on an ABI 7500 Fast instrument (Thermo Fisher Scientific) using the standard mode with the following conditions: one cycle of 50°C for 5 min, one cycle of 95°C for 20 s, and 40 cycles of 95°C for 3 s and 60°C for 30 s. The analysis was done using an automatic baseline. A cycle threshold (Ct) of < 37 was considered positive, and a Ct ≥ 37 was considered negative for PRRSV. A NEG-CONTROL group was used for monitoring after cross-contamination. A POS-CONTROL group was included concerning accounting for any problem with the virus strain used for the challenge. The NEG-CONTROL pigs were expected to remain negative for the entire study duration and served as a control for possible unintended cross-contaminations between the pig rooms. The POS-CONTROL pigs were expected to show higher viremia and nasal shedding as compared to vaccinated pigs.

### 2.12 Necropsy

All pigs were humanely euthanized at 10 dpc by pentobarbital overdose and necropsied. The severity of macroscopic lung lesions was scored as a percentage of the lung surface affected by lesions by a pathologist (PGH) blinded to the treatment status of the pigs. Tissues (lungs and tracheobronchial lymph nodes) were collected in 10% neutral buffered formalin for histopathology, and lungs were scored for the severity of interstitial pneumonia ranging from 0 (normal) to 6 (diffuse, severe), as described by Halbur et al. (21). The PRRSV antigen load in lung tissues was assessed using immunohistochemistry (IHC) (22), with scores ranging from 0 (no PRRSV present) to 3 (large levels of antigen diffusely distributed) by a pathologist (PGH) blinded to the treatment status of the pigs.

### 2.13 Statistical analysis

Means and SEM were calculated using R v 4.3.3. A *p* < 0.05 was considered significant. A type 1 one-way analysis of variance (ANOVA) was used for pairwise comparison of the average daily weight gain. The analysis for serology over time and PRRSV RNA in serum or nasal swabs was conducted using linear mixed-effects models with “Treatment”, “Day”, and “Treatment^*^Day” as fixed effects and “Pig ID” as the random effect. The model was fitted using the “lme4” package v.1.1-35.1 in R v.4.3.3. Thereafter, *post-hoc* pairwise comparisons (with the Tukey's method for adjustment) among treatment groups on each day were conducted using estimated marginal means via the ‘emmeans' package v.1.10.0 to determine whether the groups significantly differed from each other in terms of their effects on the serology. Gross lesions, interstitial pneumonia, and PRRSV IHC scores were analyzed using a non-parametric ANOVA (the Kruskal–Wallis rank sum test).

### 2.14 Microbiome analysis

Ileum content sample collections at 6 (vaccination), 9 (dpv 21), 10 (challenge), and 12 (necropsy) weeks of age were used for the analysis of the microbiome based on 16S rRNA gene V4 region amplicon diversity analysis using the Illumina MiSeq platform and mothur MiSeq Standard operating protocol. A phyloseq object (https://joey711.github.io/phyloseq/) generated from the mothur https://mothur.org/ was used in the Microbiome package in R (https://microbiome.github.io/tutorials/) to analyze changes to the beta, alpha diversity, taxonomic taxonomic composition including the core microbiome trajectories of the ileum. We then used the permutational multivariate analysis of variance (PERMANOVA) to attribute any structural variations in the ileum microbiome to our experiments. To quantify multivariate community-level differences among groups, we used the statistical analyses in the Microbiome package in R (https://microbiome.github.io/tutorials/), including PERMANOVA (23). Canonical analysis of principal coordinates or “CAP” was used for analysis of principle components (https://esajournals.onlinelibrary.wiley.com/doi/full/10.1890/0012-9658%282003%29084%5B0511%3ACAOPCA%5D2.0.CO%3B2).

## 3 Results

### 3.1 Recovery of the pigs and transport back to the pig facilities

Once the surgery was completed for a given pig, it was transported back to the research facility and placed on a rubber mat under a heat lamp. The recovery process was closely monitored by Laboratory Animal Resources (LAR) technicians. Overall, the surgeries went well, with the first group of pigs back in their pens and awake and active within 2–3 h. Images of the pigs right after being returned to their pens are provided in Supplementary Figure S4.

### 3.2 Clinical signs and post-surgery observations

Within 3–4 h after surgery, most pigs were active and alert and eating feed. Pigs that developed complications and were treated and/or euthanized are summarized in Supplementary Table S5. During the days after cannulation surgery, 4 out of 15 pigs developed clinical signs and had to be euthanized (a POS-CONTROL pig, a VAC-ANTI-PRRSV pig, a VAC-PRRSV pig, and a VAC-PRO-PRRSV pig), reducing treatment group size from 3 to 2 pigs. Necropsy of the four pigs euthanized due to complications from surgery revealed peritonitis. This peritonitis was associated with the end of the cannula eroding or tearing through the intestine rather than leakage at the purse-string at the enterotomy site. This erosion or tearing may have been due to the flange of the cannula being too large for the diameter of the intestines and/or too much movement of cannulas associated with threads not keeping the cannula flush with the abdominal wall. Thus, a grommet and spacer were added to the canulas of several pigs post-operatively (Supplementary Figure S6).

### 3.3 Average daily gain

The average daily gain is summarized in Table 2. There were no significant differences among groups. Upon arrival, the pigs' weights ranged from 5.0 to 6.6 kg. At necropsy, the lightest pig weighed 34.9 kg, whereas the heaviest pig weighed 43.4 kg.

**Table 2:** Average daily weight gain of the pigs during different phases in kg ± SEM (2 pigs per group).

**Group**	**Number of pigs**	**Arrival to vaccination**	**Arrival to necropsy**	**Vaccination to necropsy**
NEG-CONTROL	2	0.320 ± 0.03	1.195 ± 0.02	1.453 ± 0.01
POS-CONTROL	2	0.319 ± 0.03	1.186 ± 0.12	1.44 ± 0.05
VAC-ANTI-PRRSV	2	0.313 ± 0.01	1.235 ± 0.09	1.521 ± 1.04
VAC-PRO-PRRSV	2	0.273 ± 0.06	1.105 ± 0.04	1.369 ± 0.01
VAC-PRRSV	2	0.306 ± 0.03	1.265 ± 0.06	1.575 ± 0.06

There were no significant differences among computed group weight gain (P < 0.05) between arrival and vaccination (21 days), arrival and necropsy (61 days), and vaccination and necropsy (40 days).

### 3.4 Serology response to vaccination and challenge

**Figure 3:**
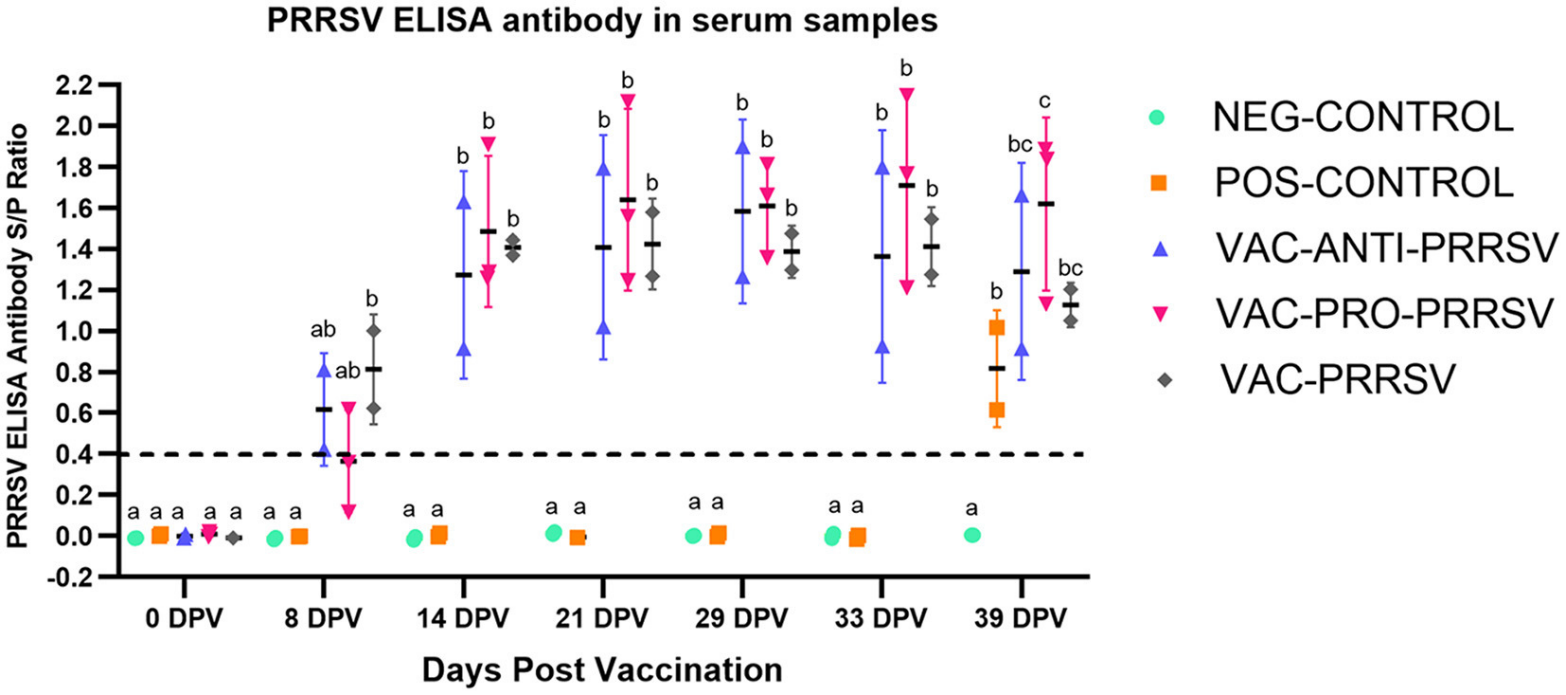
Antibody response to PRRSV at different time points including at 0-day post-vaccination (0 dpv), challenge (29 dpv), and necropsy (39 dpv, 10 days after challenge). Different superscripts (^a, b, c^) for a given day indicate significant (*P* < 0.05) differences in the antibody levels among the groups.

All pigs tested negative for anti-PRRSV antibodies at arrival and on the vaccination day (0 dpv). NEG-CONTROL pigs remained antibody-negative throughout the study, while all vaccinated pigs seroconverted to PRRSV ~1 week after vaccination (Figure 3). While the VAC-PRO-PRRSV group had numerically the highest level of seroconversion, this was not significantly different from the VAC-ANTI-PRRSV and the VAC-PRRSV groups. The non-vaccinated POS-CONTROL pigs only seroconverted at the time of necropsy, 10 days after the challenge.

### 3.5 PRRSV viremia and nasal shedding after challenge

**Figure 4:**
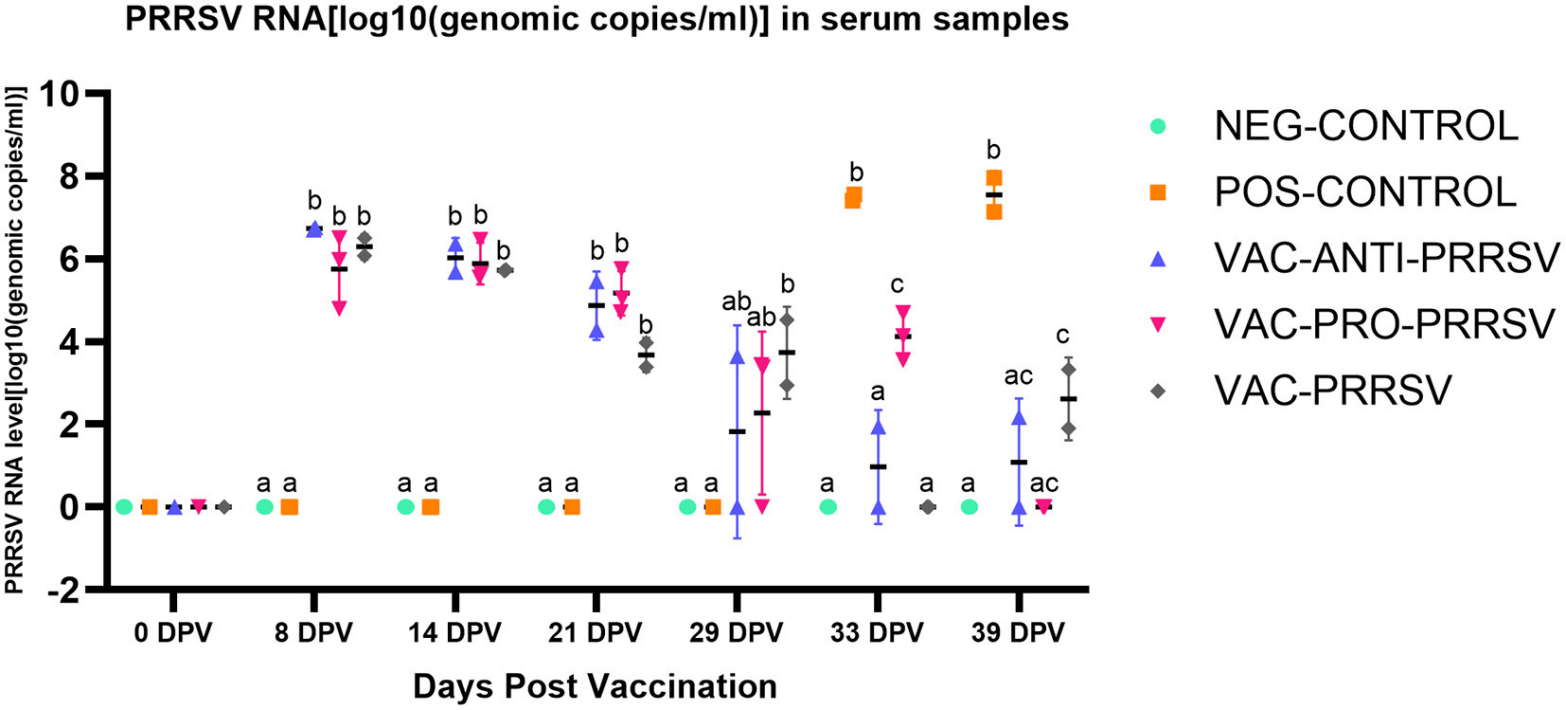
PRRSV viremia (serum) at different days post-vaccination (dpv) including initial vaccination at 0 dpv, challenge (29 dpv, 0 day post-challenge), and necropsy (39 dpv, 10 days post-challenge). Different superscripts for a given day ^(a, b, c)^ indicate significant (*P* < 0.05) differences among groups.

**Figure 5:**
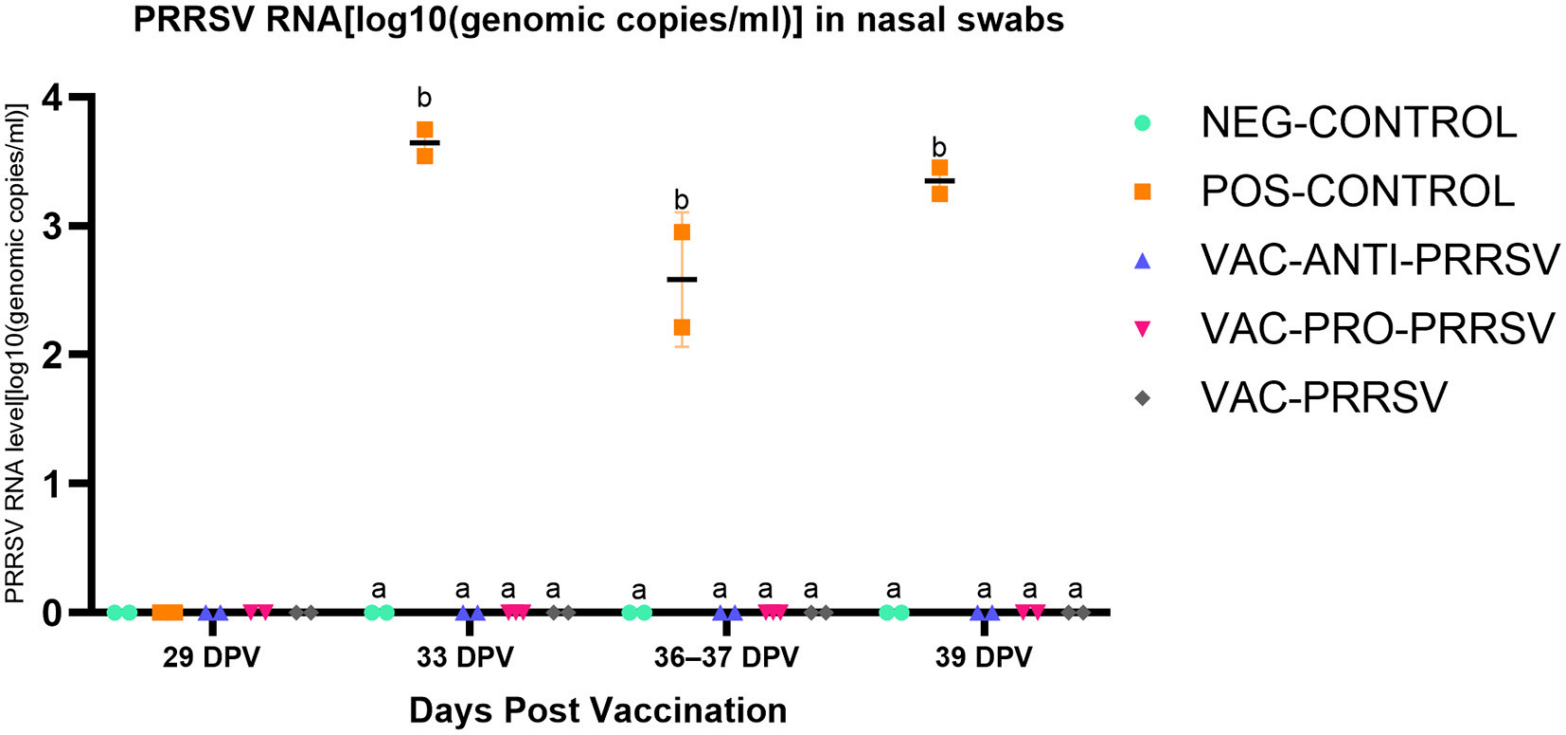
PRRSV shedding (nasal swabs) at different days post-vaccination (dpv). Samples tested included challenge at 29 dpv (corresponding to 0 day post-challenge [0 dpc]), and 33 dpv (4 dpc), 36–37 dpv (7-8 dpc), and 39 dpv (= necropsy day; 10 dpc). Different superscripts for a given day ^(a, b)^ indicate significant (*P* < 0.05) differences among groups.

The NEG-CONTROL pigs remained negative for PRRSV RNA in both serum and nasal swab samples throughout the study (Figures 4, 5). The vaccinated pigs (VAC-ANTI-PRRSV, VAC-PRO-PRRSV, and VAC-PRRSV groups) became viremic, starting with 1 week after vaccination. In these groups, the highest viremia level was detected at 8 dpv, and PRRSV genomic copies in serum started to decline afterward. There were no significant differences regarding the viremia levels between the three vaccinated groups at each time point during 8–29 dpv. After the challenge, the POS-CONTROL pigs had the highest viremia level among all groups at 33 dpv (4 dpc) and 39 dpv (10 dpc), while the viremia levels among the vaccinated groups were overall not significantly different (Figure 4). In nasal swabs, RNA-positive samples were only detected in the POS-CONROL pigs post-challenge (Figure 5). Nasal shedding is important in the transmission of PRRSV, and it appears that the shedding was blocked by vaccination, regardless of treatment at vaccination.

### 3.6 Macroscopic and microscopic lesions and PRRSV IHC results in lung tissues

At necropsy, macroscopic lung lesions were characterized by multifocal consolidation and a dark red color (Table 3). Microscopic lesions were characterized by mild-to-moderate type 2 pneumocyte hypertrophy and hyperplasia and mild lymphocytic septal infiltration. PRRSV antigen was demonstrated by IHC associated with lung lesions in the POS-CONTROL pigs (score of 2) and in one VAC-ANTI-PRRSV pig. No PRRSV antigen was detected in the lungs of the other pigs. No significant differences were observed among the groups for gross lung lesion scores, the severity of interstitial pneumonia, or PRRSV IHC scores.

**Table 3:** Group means (2 pigs per group) for macroscopic lung lesions, interstitial pneumonia, and PRRSV antigen in lung tissues, as determined by IHC.

**Group**	**Pig#**	**Gross lung lesions (Score: 0–100%)**	**Interstitial pneumonia (Score: 0–6%)**	**PRRSV IHC (Score: 0–3)**
NEG-CONTROL	2	0/2 (0)*^*a*^*	1/2 (0.5 ± 0.5)*^*a*^*	0/2 (0)*^*a*^*
POS-CONTROL	2	2/2 (17.5 ± 12.5)*^*a*^*	2/2 (2 ± 1.0)*^*a*^*	2/2 (2 ± 0)*^*a*^*
VAC-ANTI-PRRSV	2	0/2 (0)*^*a*^*	1/2 (0.5 ± 0.5)*^*a*^*	1/2 (0.5 ± 0.5)*^*a*^*
VAC-PRO-PRRSV	2	0/2 (0)*^*a*^*	2/2 (1 ± 0)*^*a*^*	0/2 (0)^a^
VAC-PRRSV	2	0/2 (0)*^*a*^*	2/2 (1 ± 0)*^*a*^*	0/2 (0)*^*a*^*

Different superscripts ^a, b^ within a given column indicate significant (P < 0.05) differences among the groups.

### 3.7 Ileum microbiome over time

**Figure 6:**
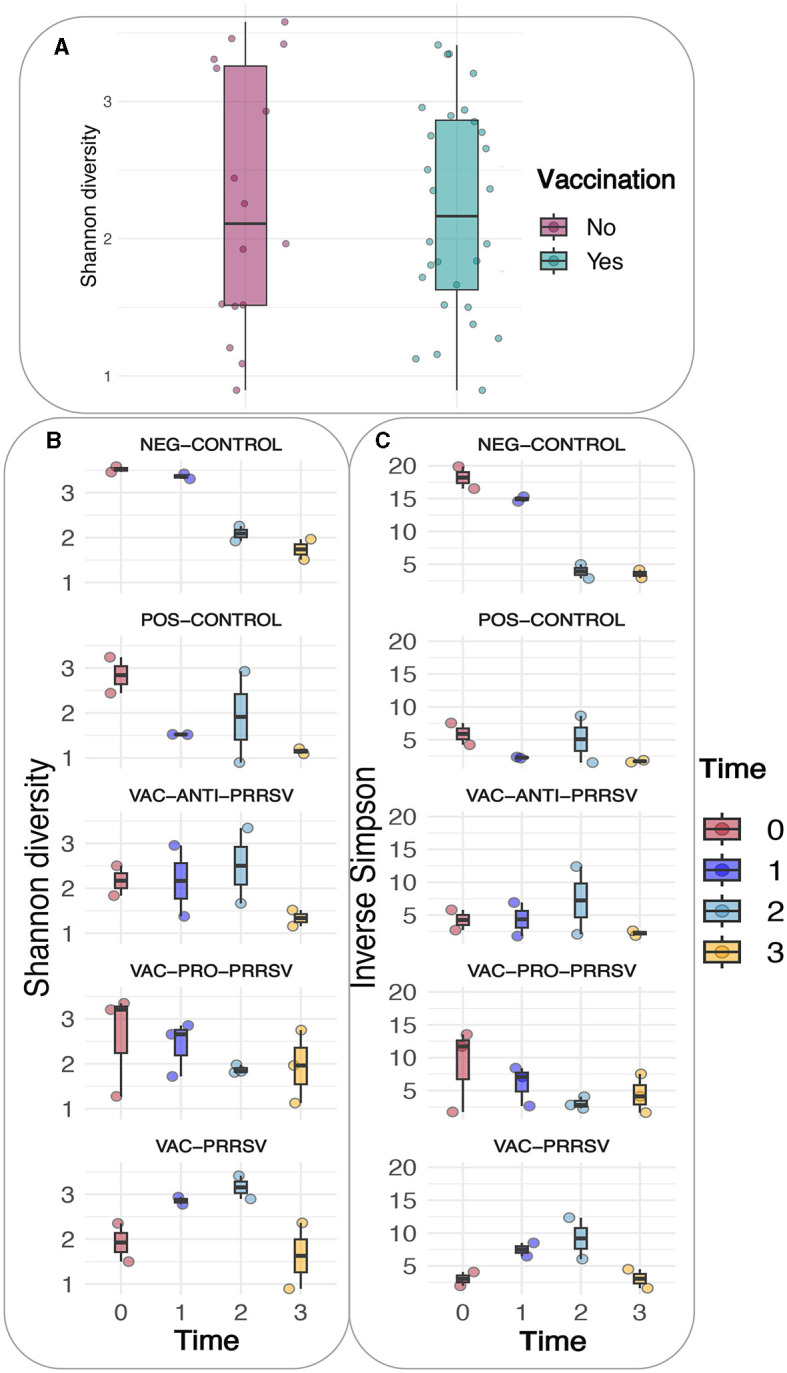
**(A)** Shannon diversity based on vaccination status. **(B)** Shannon diversity of the different groups. **(C)** Inverse Simpson of the different groups.

A comparison of alpha diversity indices, i.e., Shannon and Inverse Simpson indices, between vaccinated and non-vaccinated animals is shown in Figure 6A. Although the vaccinated pigs had a slightly lower variation yet higher means of the alpha diversity index when compared to the non-vaccinated pigs, this difference was not statistically significant. However, when both the treatment and time were considered as shown (Figures 6B, C), clear trends became evident in both indices, including the richness, rare taxa (Shannon), evenness, and dominant taxa (Simpson). The major trends observed included a gradual decline in the NEG-CONTROL group, a rapid decline in the POS-CONTROL group, an initial increase followed by a decline toward the end in the VAC-ANTI-PRRSV group, a gradual decline with high variations in the VAC-PRO-PRRSV group, and a rapid increase followed by a decline toward the end in the VAC-PRRSV group.

**Figure 7:**
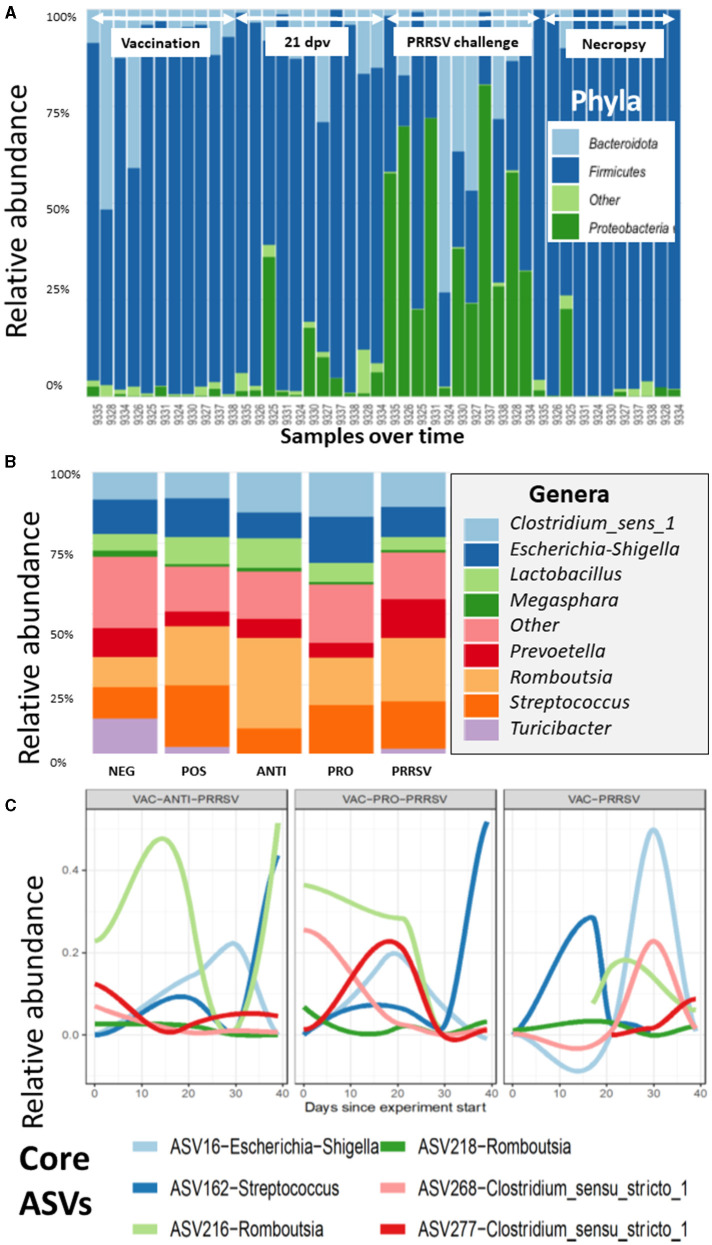
**(A)** The variation of the taxa of all the samples arranged by time at the phylum level, as demonstrated for the Proteobacteria, which have a distinctive shift over time. Time points and arrows on top indicate the different sample collections. Day post vaccination is indicated by dpv. **(B)** The contribution of major bacterial genera is compared in the different treatment groups. **(C)** The composition of the core microbiome is tracked over time, and it appears that the vaccinated groups show clear differences in the core trajectory over time.

The taxonomic composition showed a temporal change at the phylum level with a notable surge in Proteobacteria midway through the experimental period (Figure 7A). At the genus level, these shifts are predominantly driven by the ileal-adapted *Romboustia, Streptococcus*, and *Clostridium* (Figure 7B). Investigating this trajectory of the core genera at Amplicon Sequence Variants (ASV) revealed that two variants of *Romboustia* and *Clostridium* exhibit distinct abundance in the treatment groups of vaccinated pigs, as shown in Figure 7C. This observation was validated by analysis of composition of microbiomes (ANCOM) at the genus (L6) level, which shows three genera that were differentially abundant among the treatment groups over time: Streptococcus, Turicibacter, and Clostridium_sensu_stricto_6. This is evidence that they may act as beneficial bacteria in the gut flora (24–26). Both the NEG-CONTROL group and the VAC-PRO-PRRSV group had relatively higher abundance levels of Streptococcus over time compared to the NEG-CONTROL group and the VAC-ANTI-PRRSV group. All four groups showed the highest abundance levels of Streptococcus at the time of necropsy 10 days after the PRRSV challenge.

For Turicibacter, the NEG-CONTROL group showed a considerable increase in Turicibacter abundance from dpv 21 to dpc 10, relative to the other four groups. In addition, pigs in the VAC-ANTI-PRRSV and VAC-PRO-PRRSV groups shared a similar trend of having a low abundance of this genus relative to the other groups across the entire study. Finally, for Clostridium_sensu_stricto_6, all groups except the NEG-CONTROL group shared a similar trend in the abundance of this genus throughout the entire period of the study, including the highest abundance levels of this genus 4 weeks after vaccination (dpv 28) relative to the other time points.

Similar to the alpha indices, there were no discernible differences in the abundance at any of the taxonomic levels between the vaccinated and the non-vaccinated groups based on the ANCOM using QIIME composition.

**Figure 8:**
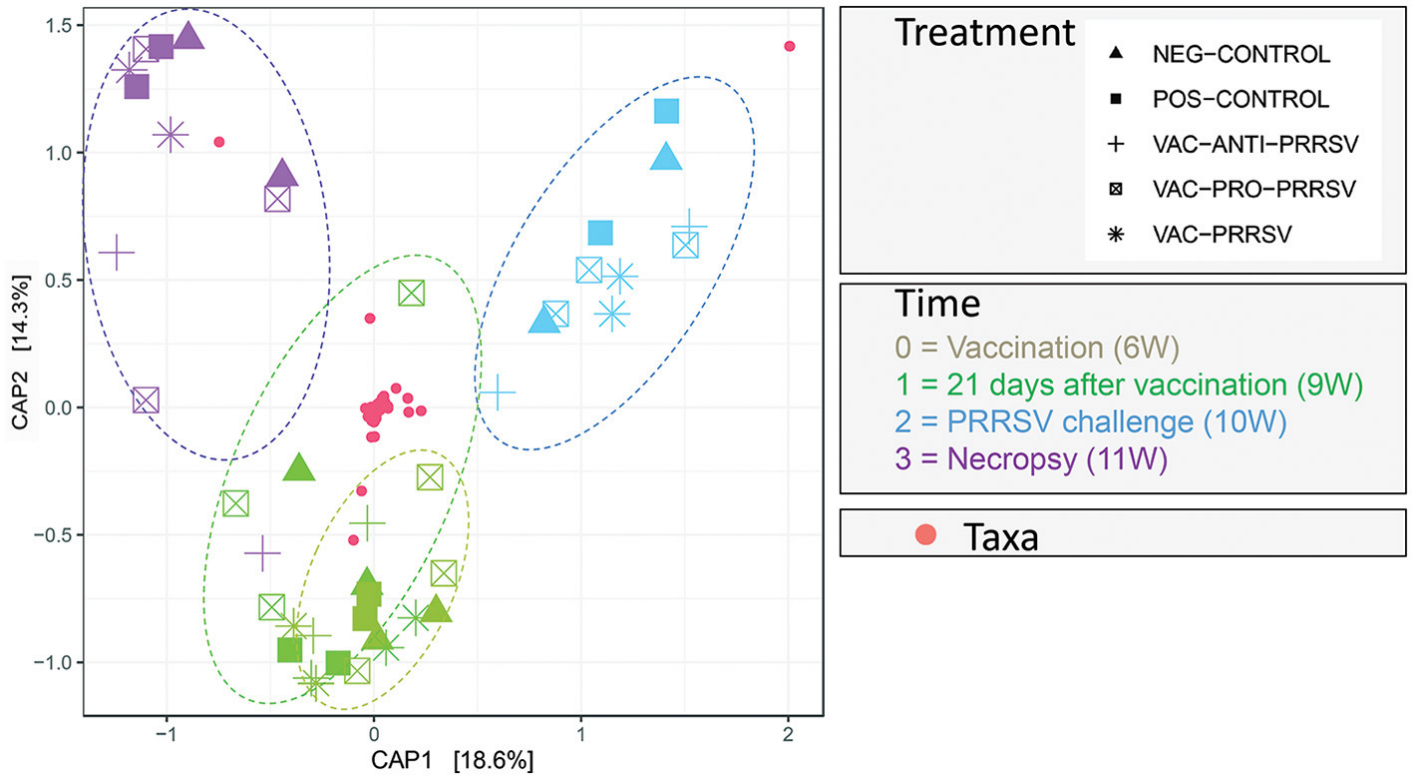
Principal Coordinate of Analysis plot showing the clustering of each pig treatment groups' microbiome by age in weeks (W).

The results in Figure 8 demonstrated a distinctive clustering of samples by time, indicating that time explained approximately 32.9% of the microbiome structural variation. When using PERMANOVA, considering the individual animal variation, it is evident that approximately 43% of the structural variation in the ileum was explained by time (36%) and treatment (7%). We used the constrained principal coordinate analysis (PCoA) here. When we constrained the PCoA by time, 32.9% of the structural variation in the microbiome was explained based on CAP1 (18.6) plus CAP2 (14.3). This was validated by the PERMANOVA (Table 4), which indicated that time explained 36.4% of the microbiome structural variation.

**Table 4:** PERMANOVA output.

**Variable**	**Degrees of freedom**	**R2**	***P*-value**
Treatment	4	0.07	0.0001
Time	3	0.36	0.0002
Residual	36	0.56	-
Total	43	1	

## 4 Discussion

The novelty of this pilot study is the description and utilization of a cannulation method in young pigs to collect small intestine contents for dynamic evaluation of the microbiota. Cannulated pigs are commonly used in nutrition studies (27–29) but are rarely utilized in infectious disease research. Currently, the pig microbiome is often investigated by sequencing rectal swabs (30, 31). However, the porcine gut microflora changes dramatically across the different gut sections; thus, the microbiome from rectal swabs is likely not representative of much of the gut microbiome, particularly the small intestines. In this study, we developed and described a cannulation method to collect small intestine contents from pigs to investigate dynamic changes in the microbiomes. Previous studies have shown that the microbiota of the small intestine is phylogenetically much less diverse than that of the colon but more dynamic (32). Future applications of this cannulation model could advance our understanding of the colonialization sites of microbes (pathogenic and nonpathogenic), the impact of feed additives, including probiotics, and the vaccines utilized to mitigate enteric and systemic diseases.

Several research groups have found a consistent difference between microbial communities of the upper and lower gastroenteric tracts. In humans, it has been shown that one community colonizes the duodenum down to the proximal ileum dominated by Pseudomonadota, Streptococcaceae, and Veillonellaceae among others, while other bacteria colonize the distal ileum down to the rectum (generally dominated by Bacteroidaceae, Lachnospiraceae, and Ruminococcaceae) (33). In our study, there were no major differences over time among bacterial genera (Figure 7). However, in Figure 7B, there is evidence of a higher abundance of *Romboutsia* in the VAC-ANTI-PRRSV group compared to the VAC-PRO-PRRSV group. In contrast, *Streptococcus* appeared more abundant in the VAC-PRO-PRRSV than in the ANTI-PRRSV group. Possible explanations for these findings include a direct impact of the treatments (probiotics and antibiotics) or intrinsic regulation of the intestinal flora balance due to other reasons. Much work remains to be done to identify essential microbes that may trigger enhanced immune responses, and such discoveries would be a major breakthrough in vaccinology and preventative medicine in animals and humans.

The impact of the gut microbiota on vaccine efficacy is still poorly understood. Under normal circumstances, vaccines for pigs are readily available and commonly effective, as evidenced by the reduction in disease spread and the reduced impact of clinical signs on both the individual pig and the herd levels. In human studies, there is an indication that the composition and function of the gut microbiota are important to overall health, as they are the key factors in modulating the immune responses to vaccination (34). The intrinsic gut microbiota is a complex accumulation of bacteria, viruses, archaea, and fungi, which likely affect humans and animals, similarly, by maintaining gastrointestinal homeostasis, regulating immune system development, metabolizing nutrients, and preventing pathogen colonization (35). In addition, microbiota could also act as a natural adjuvant, regulate host immune responses, and carry epitopes that are similar to vaccine antigens to induce cross-reaction and other ways to affect vaccine efficacy (36). Nevertheless, despite the optimistic prospects of improving gut health to enhance vaccinations, gut microbiota could also adversely affect vaccine efficacy by biasing antibody responses toward non-protective vaccine antigens similar to commensal bacterial antigens (37). At this point, larger studies on the impact of certain microbiota in pigs are lacking. We anticipate that the development and description of our model will allow researchers to identify new pathways to further improve vaccine efficacy and better understand the use of probiotic supplementation.

After cannulation surgery on 4-week-old pigs, four pigs were euthanized due to complications from surgery. However, in the future, this can likely be prevented by the use of a smaller cannula that would be less likely to result in damage to the intestinal wall and associated peritonitis. The use of older pigs may also result in fewer complications related to cannula size but may not be appropriate to investigate the microbiome in newly weaned pigs.

In this study, the limited sample size of only 2–3 pigs per group at the time of vaccination and PRRSV challenge did not provide sufficient statistical power for robust analysis and conclusions. Nevertheless, it is noteworthy that, despite having a cannulation surgery and receiving various treatments, the pigs displayed a good antibody response after vaccination. The antibody responses together with the PCR results in serum and nasal swab samples suggest that the pigs were able to mount humoral immunity successfully, thereby preventing PRRSV infection and shedding in vaccinated and challenged pigs. These findings suggest that cannulation surgery may not significantly impact the pig's reaction to infectious agents, including induction of antibody response. This evidence is promising as it supports the viability of this model for use in a large cohort of pigs, potentially enabling the detection of treatment differences.

The benefits of a healthy gut microbiome in controlling enteric infections are already widely appreciated (38–40). One of the main outcomes of this research is the development of a model that can better assess the utility of probiotics for the improvement of the efficacy of vaccines through modulation of the gut microbiome. In this context, we evaluated the effect of a probiotic on PRRSV vaccine efficacy; however, the study outcome could be applied to other viral or bacterial vaccines for pigs.

In conclusion, while microbiome studies over time have been previously performed using rectal swabs, to our knowledge, this study provides novel information on the dynamics of ileum microbiota in recently weaned pigs. The microbiome in fecal samples vs. intestinal content can vary quite dramatically, and hence, rectal swabs may not provide an accurate reflection of the microbiome in the ileum or other small intestinal sections. Although this small-scale pilot study did not allow us to conclude the impact of microbiota on PRRSV vaccination, we believe it is important to disseminate the findings from the current study using the cannulation model for the scientific community. In the future, the procedure could be further refined to result in a reliable model for longitudinal microbiome studies in recently weaned pigs.

## Data availability statement

Raw sequencing files are available from the European Nucleotide Archive BioProject number (https://www.ebi.ac.uk/ena/browser/view/PRJEB72452).

## Ethics statement

The animal study was approved by the study was conducted according to the guidelines of the Declaration of Helsinki and approved by the by the Iowa State University Institutional Animal Care and Use Committee (Approval number IACUC-21-031; Date of approval: 05-April-2021) and by the Iowa State University IBC Committee (Approval number IBC 21-019; Date of approval: 6-April-2021). Environmental enrichment was provided and independent veterinarians, not part of the research team, assessed the pigs and made decisions on welfare and euthanasia. The study was conducted in accordance with the local legislation and institutional requirements.

## Author contributions

TO: Writing – review & editing, Writing – original draft, Supervision, Funding acquisition, Project administration, Methodology, Conceptualization. PH: Investigation, Writing – review & editing, Writing – original draft, Supervision. JB: Methodology, Conceptualization, Writing – review & editing, Writing – original draft, Supervision. GR: Supervision, Writing – review & editing, Writing – original draft. HT: Methodology, Writing – review & editing, Writing – original draft. KM: Writing – review & editing, Writing – original draft, Data curation. GL: Data curation, Writing – review & editing, Writing – original draft. DZ: Writing – review & editing, Writing – original draft, Formal analysis. JZ: Formal analysis, Writing – review & editing, Writing – original draft. AM: Data curation, Visualization, Methodology, Writing – review & editing, Writing – original draft.

## References

[1] Lee J Htoo JK Kluenemann M González-Vega JC Nyachoti CM. Effects of dietary protein content and crystalline amino acid supplementation patterns in low protein diets on intestinal bacteria and their metabolites in weaned pigs raised under Different sanitary conditions. J Anim Sci. (2023) 101:skad252. 10.1093/jas/skad252 37527457 PMC10439707

[2] Pandey S Kim ES Cho JH Song M Doo H Kim S. Swine gut microbiome associated with non-digestible carbohydrate utilization. Front Vet Sci. (2023) 10:1231072. 10.3389/fvets.2023.1231072 37533451 PMC10390834

[3] Trudeau MP Mosher W Tran H de Rodas B Karnezos TP Urriola PE. Experimental facility had a greater effect on growth performance, gut microbiome, and metabolome in weaned pigs than feeding diets containing subtherapeutic levels of antibiotics: a case study. PLoS ONE. (2023) 3:e0285266. 10.1371/journal.pone.0285266 37535525 PMC10399857

[4] Melendez Hebib V Taft DH Stoll B Liu J Call L Guthrie G. Probiotics and human milk differentially influence the gut microbiome and nec incidence in preterm pigs. Nutrients. 15:2585. 10.3390/nu15112585 37299550 PMC10255242

[5] Rutjens S Vereecke N Sauer J Croubels S Devreese M. Cefquinome shows a higher impact on the pig gut microbiome and resistome compared to ceftiofur. Vet Res. (2023) 54:45. 10.1186/s13567-023-01176-8 37280708 PMC10242799

[6] Fox BE Vilander AC Gilfillan D Dean GA Abdo Z. Oral vaccination using a probiotic vaccine platform combined with prebiotics impacts immune response and the microbiome. Vaccines. (2022) 10:1465. 10.3390/vaccines10091465 36146543 PMC9504555

[7] Lenoir-Wijnkoop I Merenstein D Korchagina D Broholm C Sanders ME Tancredi D. Probiotics reduce health care cost and societal impact of flu-like respiratory tract infections in the USA: an economic modeling study. Front Pharmacol. (2019) 10:1182. 10.3389/fphar.2019.00980 31649544 PMC6798268

[8] Boge T Rémigy M Vaudaine S Tanguy J Bourdet-Sicard R van der Werf S. A probiotic fermented dairy drink improves antibody response to influenza vaccination in the elderly in two randomised controlled trials. Vaccine. (2009) 27:5677–84. 10.1016/j.vaccine.2009.06.094 19615959

[9] Zimmermann P Curtis N. The influence of probiotics on vaccine responses - a systematic review. Vaccine. (2018) 36:207–13. 10.1016/j.vaccine.2017.08.069 28923425

[10] González-Solé F Camp Montoro J Solà-Oriol D Pérez JF Lawlor PG Boylee LA. Effect of mixing at weaning and nutrient density of the weaner diet on growth performance and welfare of pigs to slaughter. Porcine Health Manag. (2023) 9:38. 10.1186/s40813-023-00334-w 37641119 PMC10464064

[11] Luo Y Ren W Smidt H Wright AG Yu B Schyns G. Dynamic distribution of gut microbiota in pigs at different growth stages: composition and contribution. Microbiol Spectr. (2022) 10:e0068821. 10.1128/spectrum.00688-21 35583332 PMC9241710

[12] Muwonge A Karuppannan AK Opriessnig T. Probiotics mediated gut microbiota diversity shifts are associated with reduction in histopathology and shedding of Lawsonia intracellularis. Anim Microbiome. (2021) 3:22. 10.1186/s42523-021-00084-6 33663618 PMC7931366

[13] Opriessnig T Gerber PF Shen H de Castro AMMG Zhang J Chen Q. Evaluation of the efficacy of a commercial inactivated genogroup 2b-based porcine epidemic diarrhea virus (PEDV) vaccine and experimental live genogroup 1b exposure against 2b challenge. Vet Res. (2017) 48:69. 10.1186/s13567-017-0472-z 29073936 PMC5659040

[14] Halbur PG Paul PS Meng XJ Lum MA Andrews JJ Rathje JA. Comparative pathogenicity of nine US porcine reproductive and respiratory syndrome virus (PRRSV) isolates in a five-week-old cesarean-derived, colostrum-deprived pig model. J Vet Diagn Invest. (1996) 8:11–20. 10.1177/104063879600800103 9026065

[15] Metzler-Zebeli BU Rosenfelder-Kuon P Brehm H Eklund M Mosenthin R. Improved simple T-cannula technique to facilitate surgery and daily skin care of growing pigs. J Anim Sci. (2020) 98:skaa091. 10.1093/jas/skaa091 32206780 PMC7135948

[16] Stein HH Shipley CF Easter RA. Technical note: a technique for inserting a T-cannula into the distal ileum of pregnant sows. J Anim Sci. (1998) 76:1433–6. 10.2527/1998.7651433x 9621950

[17] Wubben JE Smiricky MR Albin DM Gabert VM. Improved procedure and cannula design for simple-T cannulation at the distal ileum in growing pigs. Contemp Top Lab Anim Sci. (2001) 40:27–31. 11703054

[18] Radcliffe JS Rice JP Pleasant RS Apgar GA. Technical Note: Improved technique for fitting pigs with steered ileocecal valve cannulas. J Anim Sci. (2005) 83:1563–7. 10.2527/2005.8371563x 15956465

[19] Dorado-Montenegro S Lammers-Jannink K Gerrits W de Vries. S. (2023). Insoluble fibers affect digesta transit behavior in the upper gastrointestinal tract of growing pigs, regardless of particle size. J Anim Sci. 101:skad299. 10.1093/jas/skad299 37665959 PMC10651184

[20] Opriessnig T Rawal G McKeen L Filippsen Favaro P Halbur PG Gauger PC. Evaluation of the intranasal route for porcine reproductive and respiratory disease modified-live virus vaccination. Vaccine. (2021) 39:6852–9. 10.1016/j.vaccine.2021.10.033 34706840

[21] Halbur PG Paul PS Frey ML Landgraf J Eernisse K Meng X. Comparison of the pathogenicity of two US porcine reproductive and respiratory syndrome virus isolates with that of the Lelystad virus. Vet Pathol. (1995) 32:648–60. 10.1177/030098589503200606 8592800

[22] Halbur PG Andrews JJ Huffman EL Paul PS Meng XJ Niyo Y. Development of a streptavidin-biotin immunoperoxidase procedure for the detection of porcine reproductive and respiratory syndrome virus antigen in porcine lung. J Vet Diagn Invest. (1994) 6:254–7. 10.1177/104063879400600219 8068760

[23] Anderson MJ. Permutational multivariate analysis of variance (PERMANOVA). In:Balakrishnan N Everitt TC Piegorsch W Ruggeri F Teugels JL, editor. Wiley Stats Ref: Statistics Reference Online. (2017). p. 1–15. Available online at: https://onlinelibrary.wiley.com/action/showCitFormats?doi=10.1002%2F9781118445112.stat07841

[24] Guo P Zhang K Ma X He P. Clostridium species as probiotics: potentials and challenges. J Anim Sci Biotechnol. (2020) 11:24. 10.1186/s40104-019-0402-1 32099648 PMC7031906

[25] Lynch JB Gonzalez EL Choy K Faull KF Jewell T Arellano A. Gut microbiota Turicibacter strains differentially modify bile acids and host lipids. Nat Commun. (2023) 14:3669. 10.1038/s41467-023-39403-7 37339963 PMC10281990

[26] Wylensek D Hitch TCA Riedel T Afrizal A Kumar N. Wortmann E, et al. A collection of bacterial isolates from the pig intestine reveals functional and taxonomic diversity. Nat Commun. (2020) 11:6389. 10.1038/s41467-020-19929-w 33319778 PMC7738495

[27] Heyer CME Wang LF Beltranena E Rodehutscord M Zijlstra RT. Effect of increasing dietary fermentable fiber on diet nutrient digestibility and estimation of endogenous phosphorus losses in growing pigs. J Anim Sci. (2023) 101:skad204. 10.1093/jas/skad204 37335891 PMC10321371

[28] Lammers-Jannink KCM Pellikaan WF de Vries S Stigter ECA Gerrits WJJ. Standardisation of the C:N ratio in ileal digesta changes relationships among fermentation end-products during in vitro hindgut fermentation in pigs. Animal. (2023) 17:101026. 10.1016/j.animal.2023.101026 38035658

[29] Zhao S Lv L Wu T Feng Z Li Q Lei L. A combined pig model to determine the net absorption of volatile fatty acids in the large intestine under different levels of crude fiber. Animal Model Exp Med. (2023) 6:375–80. 10.1002/ame2.12329 37534602 PMC10486325

[30] Choudhury R Kleerebezem M. Assessing the impact of diet on the mucosa-adhered microbiome in piglets using comparative analysis of rectal swabs and colon content. Front Microbiol. (2022) 13:804986. 10.3389/fmicb.2022.804986 35273582 PMC8902596

[31] He Y Tiezzi F Howard J Huang Y Gray K Maltecca C. Exploring the role of gut microbiota in host feeding behavior among breeds in swine. BMC Microbiol. (2022) 22:1. 10.1186/s12866-021-02409-6 34979903 PMC8722167

[32] Choudhury R Middelkoop A Bolhuis JE Kleerebezem M. Legitimate reliable determination of the age-related intestinal microbiome in young piglets; rectal swabs and fecal samples provide comparable insights. Front Microbiol. (2019) 10:1886. 10.3389/fmicb.2019.01886 31474964 PMC6702655

[33] Jensen BAH Heyndrickx M Jonkers D Mackie A Millet S Naghibi M. Small intestine vs. colon ecology and physiology: why it matters in probiotic administration. Cell Rep Med. (2023) 4:101190. 10.1016/j.xcrm.2023.101190 37683651 PMC10518632

[34] Huang B Wang J Li L. Recent five-year progress in the impact of gut microbiota on vaccination and possible mechanisms. Gut Pathog. (2023) 15:27. 10.1186/s13099-023-00547-y 37308966 PMC10258485

[35] Jordan A Carding SR Hall LJ. The early-life gut microbiome and vaccine efficacy. Lancet Microbe. (2022) 3:e787–94. 10.1016/S2666-5247(22)00185-9 36088916

[36] Lynn DJ Benson SC Lynn MA Pulendran B. Modulation of immune responses to vaccination by the microbiota: implications and potential mechanisms. Nat Rev Immunol. (2022) 22:33–46. 10.1038/s41577-021-00554-7 34002068 PMC8127454

[37] Williams WB Liao HX Moody MA Kepler TB Alam SM. HIV-1 VACCINES. Diversion of HIV-1 vaccine-induced immunity by gp41-microbiota cross-reactive antibodies. Science. (2015) 349:aab1253. 10.1126/science.aab1253 26229114 PMC4562404

[38] Liao SF Nyachoti M. Using probiotics to improve swine gut health and nutrient utilization. Anim Nutr. (2017) 3:331–43. 10.1016/j.aninu.2017.06.007 29767089 PMC5941265

[39] Pereira WA Franco SM Reis IL Mendonça CMN Piazentin ACM Azevedo POS. Beneficial effects of probiotics on the pig production cycle: an overview of clinical impacts and performance. Vet Microbiol. (2022) 269:109431. 10.1016/j.vetmic.2022.109431 35468401

[40] Vasquez R Oh JK Song JH Kang DK. Gut microbiome-produced metabolites in pigs: a review on their biological functions and the influence of probiotics. J Anim Sci Technol. (2022) 64:671–95. 10.5187/jast.2022.e58 35969697 PMC9353353

