# Supplementary material for: Exploratory application of a cannulation model in recently weaned pigs to monitor longitudinal changes in the enteric microbiome across varied porcine reproductive and respiratory syndrome virus (PRRSV) infection statuses
Source: Front Vet Sci. 2024 Jul 19;11:1422012. doi: 10.3389/fvets.2024.1422012 (PMC11294941; doi:10.3389/fvets.2024.1422012)
Supplement: Supplementary file 1 [file Data_Sheet_1.pdf]

Supplementary Material

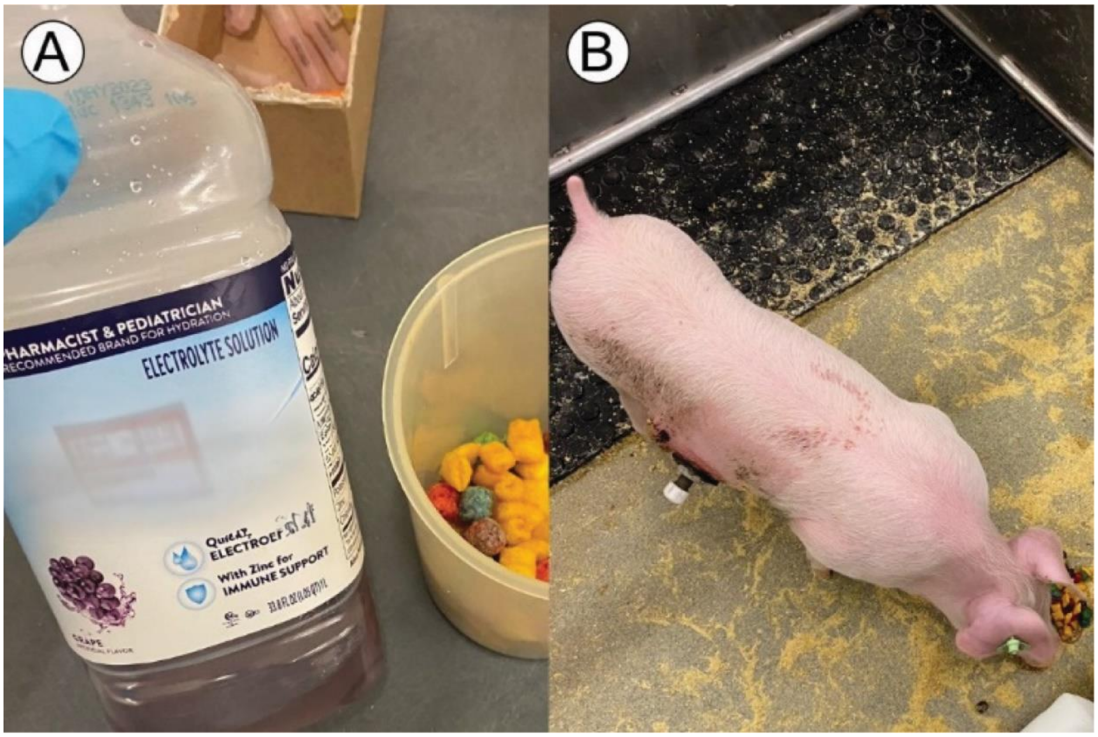

Supplementary Figure S1. Probiotic feeding. **A.** Ingredient's for administration of the probiotic including Pedialyte and Captain Crunch® cereal. **B.** A study pig readily consumes the probiotic from the floor.

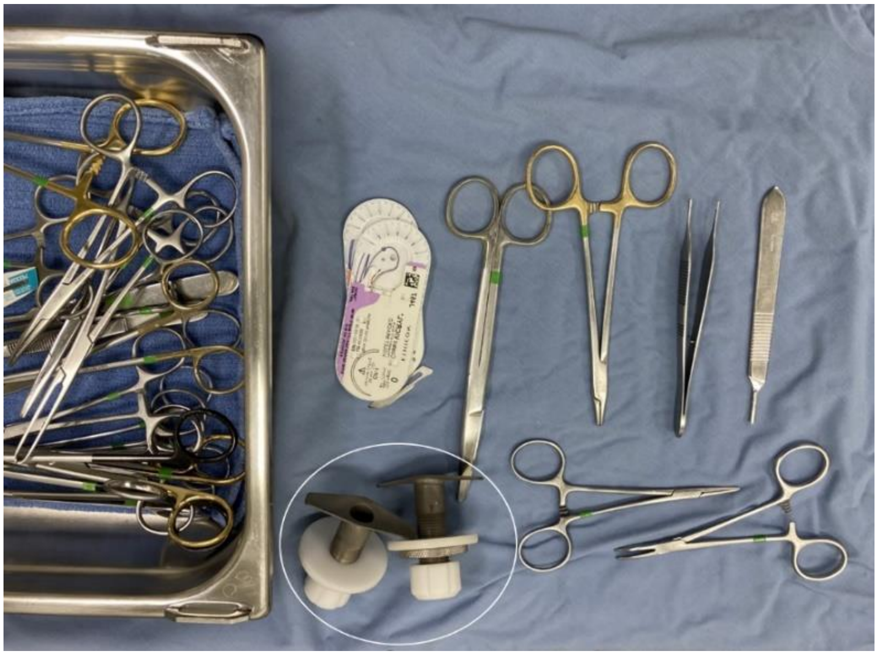

Supplementary Figure S2. Images of surgery equipment including cannula (white circle).

Supplementary Information S3. Cannulation surgery with details of the surgery procedure.

The pig was positioned in left lateral recumbency and the right paralumbar fossa (flank) was clipped, aseptically prepared, and draped routinely. A vertical incision approximately 8 cm in length was made cantered in the paralumbar fossa, 4 cm ventral to the transverse spinal processes, and 6 cm caudal to the last rib a vertical incision approximately 6-8 cm in length was made via sharp incision with a #10 scalpel blade, with the incising of the skin, subcutaneous tissues, external and internal abdominal oblique, and transversus abdominis muscles. The peritoneum was tented with forceps and opened using Mayo scissors, the length of the body wall incision. The cecum was exteriorized, and the ileocecal fold was identified and traced to the ileum. A segment of the ileum (approximately 10-15 cm in length) was exteriorized and isolated from the abdomen using lap sponges and huck towels, while the cecum and other exteriorized viscera were replaced into the abdomen. Ileal contents were gently milked into the cecum aborally as well as orally into the proximal ileum/distal jejunum, and sterile bobby pins were placed across the ileal segment on both ends to serve as ‘Doyen’ forceps to ensure peristalsis did not drive ingesta into the surgical site. Along the anti-mesenteric side, a purse-string suture was placed using a 2-0 polydioxanone (PDS) suture before incision into the lumen. The anti-mesenteric side of the ileum was incised with a #10 surgical scalpel blade, approximately 1.5 - 2 cm in length, and an intestinal T-cannula was placed into the lumen. The initial purse string suture was tightened and secured. A second purse-string suture was placed in a similar fashion. The site was checked for leaks around the cannula. A small full-thickness stab incision, cranial to the abdominal incision and approximately 1cm caudal to the last rib, was made through the body wall to tunnel and exit the cannula to the exterior. The cannula was exited through the body wall while ensuring that there were no intestinal loops trapped between the cannulated segment and the peritoneum, and that the cannula flange was in an appropriate orientation. The cannula was secured externally with a washer, and the screw-top cap was tightened using the manufacturer’s system (ring/gasket and threaded top). The body wall was closed routinely, as follows. The peritoneum was closed with 2-0

PDS in a simple continuous pattern. Using 0 PDS, the transversus, internal abdominal oblique, and external abdominal oblique muscles were closed in a single layer using a continuous suture pattern. The skin was closed in a continuous horizontal mattress pattern using 0 PDS. A topical spray wound bandage (AluSpray™) was applied to the surgical site closure prior to recovery.

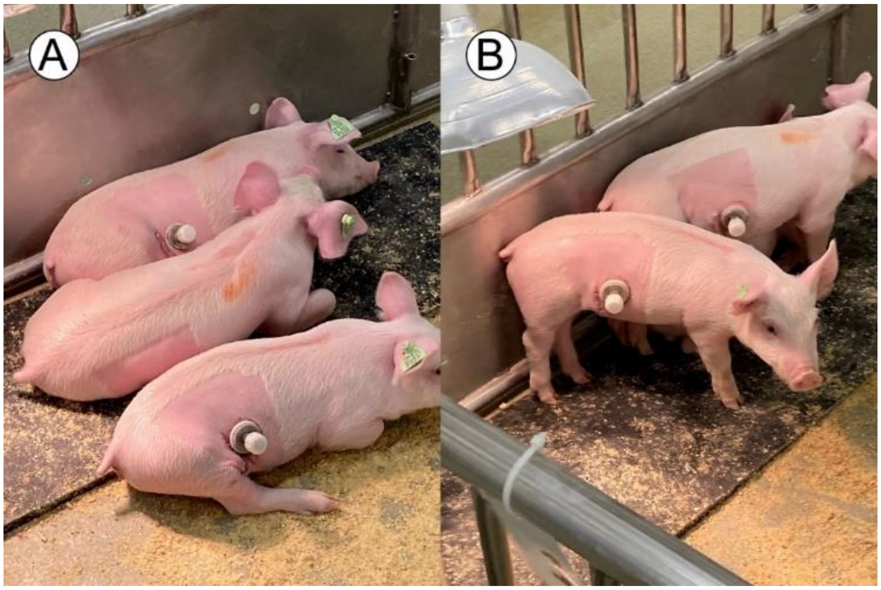

Supplementary Figure S4. Cannulated pigs recovering from surgery.

Supplementary Table S5. Clinical findings and necropsy findings of pigs that were euthanized. If a pig is not mentioned it indicates it was healthy, in normal shape and had a normal rectal temperature.

**Day post-surgery**	**Clinical findings**	**Necropsy findings/comments**
**1**	While most pigs looked good, two pigs, A VAC-ANTI-PRRSV (ID: 9332) and a POS-CONTROL pig (ID: 9329) were identified that were a bit lethargic. Rectal temps were within normal range.	It was decided to treat all pigs one more day with Naxcel (Zoetis). The two lethargic pigs were treated with Banamine (Merck Animal Health). It was further determined that unless necessary, pigs are not to be picked up or stressed by manipulating in a way that may cause problems with the cannula internally or the external incision sites.
**2**	A POS-CONTROL pig (ID: 9329) appeared less active all other pigs.	
**3**	Pig 9329 became hypothermic, lethargic and reluctant to stand and it was euthanized.	Diffuse, generalized fibrinous peritonitis, with extensive purulent exudate around ileal cannula site. The two purse string sutures were intact and holding. A 1-2mm gap along one side was observed. Likely the suture pulled through one side of the intestinal wall, albeit still holding but allowing a gap between cannula and intestinal lumen, allowing leakage of intestinal contents.
**4**	A VAC-ANTI-PRRSV pig (ID: 9332) showed separation of the cannula markedly from the body wall. Suspect cannula has become dislodged internally and/or tearing of the ileal wall. Abdomen in taught and pendulous - likely abdominal effusion present. Large amount of fluid vomitus (bile, minimal feed) was observed.	Localized septic peritonitis, full-thickness rupture ileal wall. Both purse string sutures were intact and sealed, however a moderate amount of fibrin ringing the location between cannula and body wall. The cannula flange at the aboral end tore through (full thickness) the intestinal wall antemortem. Fibrin along edges and it was attempting to wall itself off along the body wall and nearby viscera.
**5**	A VAC-PRO-PRRSV pig (ID: 9333) was found dead early in the morning.	There was localized fibrinous peritonitis and purple discoloration of skin and a mild amount of swelling of the abdominal closure. Normal appearance of scant free fluid in the abdomen, along with normal appearance of small intestinal serosa. Anti-mesenteric aspect of ileum overlying cannula flange, approximately 1.5 cm from purse-string suture, a 0.5 mm area of fibrin necrotic exudate with fibrinous adhesion to body wall. The purse string sutures were in place and fibrinous ring around cannula and body wall present.

**6**	For a VAC-PRRSV pig (ID: 9336) the cannula slightly pulled away from side. This was fixed making a grommet which worked well (S 6A).	
**7**	For the VAC-PRRSV pig 9336 the grommet had fallen off over night; the cannula has separated further from abdomen wall and the pig vomited green grainy vomit. The pig was up and ate a little before laying down. Two other pigs, a VAC-PRO-PRRSV pig (ID: 9325) and the VAC-PRO-PRRSV pig (ID: 9336) had slightly shifted cannulas. Cannula sites were cleaned and applied ointment. A spacer for the miss fitting cannulas was designed (S6 B) and the area around the canula was treated with topical SSD (1% silver sulfadiazine)	
**8**	The decision was made to euthanize a VAC-PRO-PRRSV pig (ID: 9336).	The carcass was moderately thin as evidenced by appearance of shoulder blades and spinal processes. The abdomen was moderated distended. Semisolid feces were present in the rectum. Necropsy demonstrated moderate, locally extensive fibrinous peritonitis with adhesion of the small intestines to the body wall around the cannula. The cannulated terminal ileum was intact, the intestinal lumen was patent and evidence of tearing of the ileum tissue around the cannula was lacking. The small intestine was segmentally dilated and filled with gas and fluid. No lesions were observed in other organ systems. Gross lesions suggest peritonitis likely related to the external wound around the cannula.
**50**	A VAC-PRO PRRSV pig (ID: 9325) was euthanized due to poor overall prognosis and declining overall body index score with visible scapula and vertebrae.	Abdomen moderately distended. Semisolid feces in the rectum. Moderate to severe locally extensive fibromas and peritonitis with adhesions to the small intestines. No apparent tear in the ileum. Dehiscence of the deep purse string suture.
**55**	Planned necropsies on the remaining pigs.	Presence and severity of abdomen and cannula adhesions:<br>2/9: no lesions<br>3/9: mild canula<br>1/9: moderate<br>3/9: severe

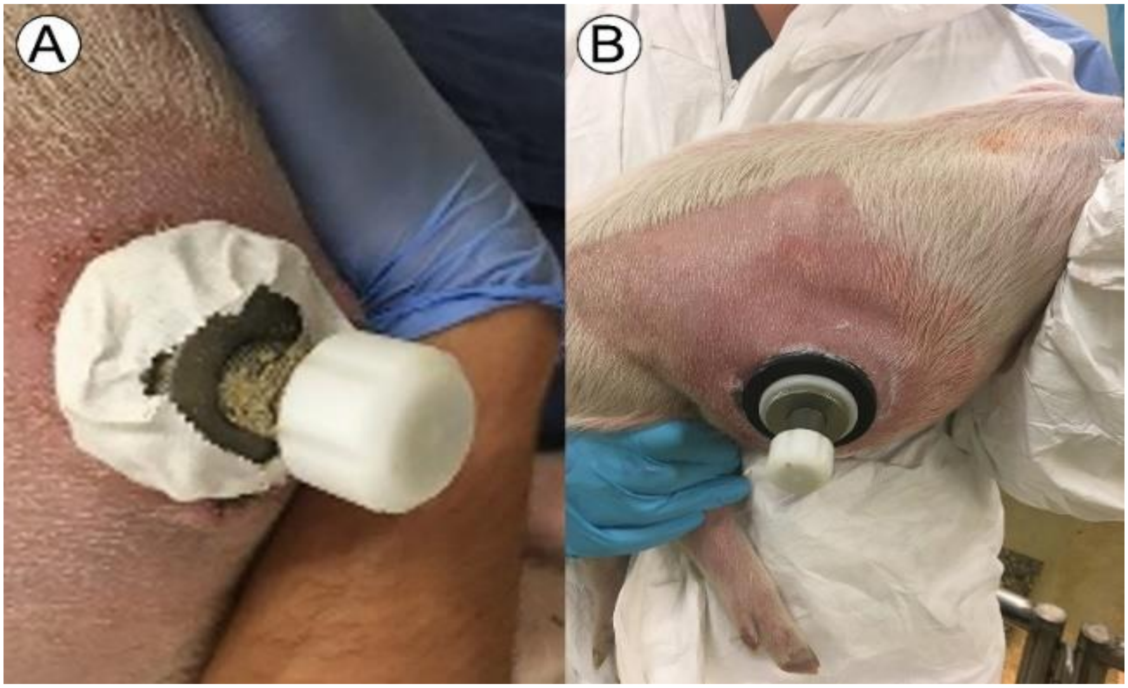

Supplementary Figure S6. A. Grommet on the pig. **B.** Spacer designed to fit the cannula.
